# SARS-CoV-2 variants retain high airborne transmissibility by different strategies

**DOI:** 10.1038/s44298-025-00120-1

**Published:** 2025-05-01

**Authors:** Jie Zhou, Ksenia Sukhova, Rebecca Frise, Laury Baillon, Jonathan C. Brown, Thomas P. Peacock, Wilhelm Furnon, Vanessa M. Cowton, Arvind H. Patel, Massimo Palmarini, Wendy S. Barclay

**Affiliations:** 1 Department of Infectious Disease, Imperial College London, London, UK; 2 The Pirbright Institute, Woking, Surrey, UK; 3 MRC-University of Glasgow Centre for Virus Research, Glasgow, UK

**Keywords:** SARS-CoV-2, Viral transmission

## Abstract

SARS-CoV-2 variants evolve to balance immune evasion and airborne transmission, yet the mechanisms remain unclear. In hamsters, first-wave, Alpha, and Delta variants transmitted efficiently via aerosols. Alpha emitted fewer viral particles than first-wave virus but compensated with a lower infectious dose (ID_50_). Delta exhibited higher airborne emission but required a higher ID_50_. A fall in airborne emission of infectious Delta virus over time after infection correlated with a decrease in its infectivity to RNA ratio in nasal wash and a decrease in contagiousness to sentinel animals. Omicron subvariants (BA.1, EG.5.1, BA.2.86, JN.1) displayed varying levels of airborne transmissibility, partially correlated with airborne emissions. Mutations in the non-spike genes contributed to reduced airborne transmissibility, since recombinant viruses with spike genes of BA.1 or JN.1 and non-spike genes from first-wave virus are more efficiently transmitted between hamsters. These findings reveal distinct viral strategies for maintaining airborne transmission. Early assessment of ID50 and aerosolized viral load may help predict transmissibility of emerging variants.

## Introduction

Despite the implementation of stringent control measures as the COVID-19 pandemic emerged and the success of new vaccines implemented with unprecedented speed, SARS-CoV-2 rapidly achieved global circulation and continues to cause respiratory disease around the world 5 years later^1^. The primary factor contributing to the continued widespread success of SARS-CoV-2 is the plasticity of the Spike protein that has enabled the continuous emergence of antigenic variants that partially escape predominant population immunity whilst maintaining the ability to transmit efficiently via airborne routes^2^. However, non-spike mutations likely contribute to viral adaptation, particularly in the nucleocapsid protein (N) and open reading frames (ORF)9b and ORF6—all established innate immune antagonists that enable immune evasion and enhance transmission potential^2–4^.

For a virus to be effectively transmitted via the airborne route, it must replicate efficiently within hosts, become aerosolised, maintain infectiousness while airborne, be inhaled by susceptible individuals, reach appropriate target cells, evade both innate and adaptive immune responses, establish a productive infection in the new host, and initiate a subsequent transmission cycle^5^. Although the capacity for airborne transmission of SARS-CoV-2 is well recognised, comprehensive studies comparing and characterising the airborne transmission dynamics of SARS-CoV-2 variants remain limited.

Hamsters are considered the gold standard model for studying SARS-CoV-2 transmission due to their manageability, natural susceptibility to the virus and its variants, and because they exhibit a range of clinical signs that closely mimic human disease^6^. Previous studies have demonstrated that pre-Omicron SARS-CoV-2 variants are transmitted efficiently via direct and indirect contact in the hamster model^6–10^. However, there remains a gap in understanding the nuances of transmission and how it may differ between variants, such as the virus’s aerosolisation capacity and stability in the air. We previously developed a unique custom-made apparatus, the Infectious Virus Transmission Tunnel (IVT), capable of quantifying airborne infectious viral emissions at three different distances (30 cm, 60 cm, and 90 cm). Detection of infectious virus in this apparatus has been shown to correlate with airborne transmissibility for influenza virus in the ferret model^11^.

Since its emergence in late 2021, descendants of the Omicron variant of SARS-CoV-2 have quickly replaced previous lineages. The Omicron variants are genetically and antigenically distinct from previous variants, leading to significant evasion of neutralising antibody responses and alterations in transmissibility and pathogenicity^10,12,13^. Despite their generally reduced pathogenicity in both humans and animal models compared to previous variants of concern (VoC), the continuous immune pressure from a population with pre-existing exposure from prior infections and vaccination has constantly driven the emergence of novel Omicron lineages with altered antigenic properties, in a process similar to antigenic drift in influenza. Omicron genetic changes are predominantly observed in the spike protein, which mediates receptor binding and viral entry, but also occur in non-spike regions of the genome and impact innate immune control^2,3^. As a participant in the UK Genotype-to-Phenotype Consortium, we routinely received clinical samples and isolated the virus from these samples. We then utilised the hamster model to assess their airborne transmissibility and ability to cause breakthrough infections^10^.

In this study, we used the hamster model to characterise the airborne transmission of pre-Omicron variants and Omicron subvariants. We also characterised their airborne viral emission. Additionally, we calculated the ID_50_ of the Alpha and Delta variants to investigate the different strategies SARS-CoV-2 has evolved to maintain efficient airborne transmissibility. The timing and duration of contagiousness in Delta-inoculated hamsters were assessed using sentinel hamsters and correlated with capacity for airborne viral emission. Furthermore, to understand the role of spike and non-spike genes in altering airborne transmissibility, we generated recombinant viruses featuring a wildtype Wuhan-Hu-1 backbone with the either the Omicron BA.1 or JN.1 Spike.

## Results

### Pre-Omicron SARS-CoV-2 variants are transmitted efficiently between hamsters via the airborne route

**Fig. 1:**
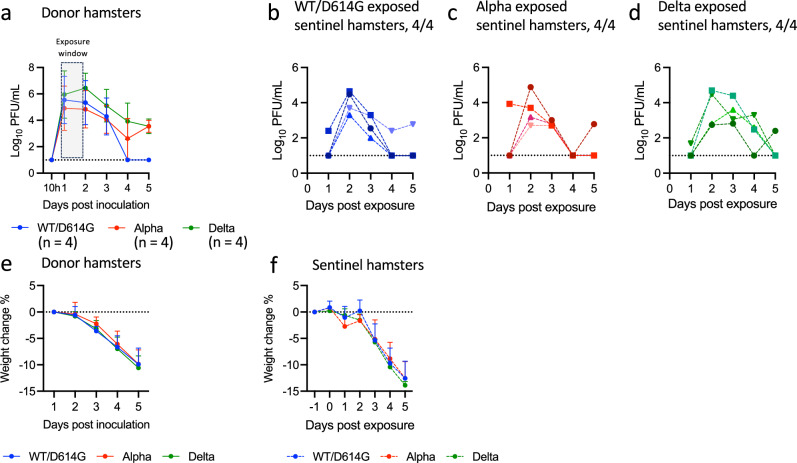
SARS-CoV-2 pre-Omicron variants, including Wildtype with D614G (WT/D614G), Alpha, and Delta variants, are transmitted efficiently in hamsters via the airborne route. Donor hamsters were inoculated intranasally with 100 PFU of first wave wildtype SARS-CoV-2 with D614G (WT/D614G) (*n* = 4), Alpha (*n* = 4), or Delta variant (*n* = 4). On day 1 post-inoculation (dpi), each donor hamster was housed on one side of a custom-made ISO-cage, while a naïve sentinel hamster was housed on the opposite side, separated by a divider consisting of two perforated metal panels spaced 1 cm apart. After 24 h of exposure, the sentinel hamsters were removed and housed individually. All hamsters were sampled daily via nasal wash, and virus titres were determined by plaque assay, with a detection limit of 10 PFU/mL (dotted lines). **a** Viral shedding in nasal washes from donor hamsters, mean and standard deviation (SD) are shown. **b**–**d** Viral shedding of sentinel hamsters exposed to WT/D614G (**b**), Alpha (**c**), or Delta donor (**d**). Each line represents the viral shedding dynamic of an individual sentinel hamster. Weight change of donor (**e**) and sentinel hamsters (**f**), mean and SD are shown.

To investigate the airborne transmission of SARS-CoV-2, donor hamsters were inoculated intranasally with 100 PFU of the first wave wildtype SARS-CoV-2 with D614G mutation on the spike protein (WT/D614G), or the same dose of Alpha and Delta variants. To confirm that no residual inoculum contributed to onward transmission, we collected nasal wash samples 10 h post-inoculation, and no infectious virus was detected by plaque assay (Fig. 1a). On day 1 post-inoculation (dpi), each donor hamster was housed on one side of a custom-made ISO-cage, while a naïve sentinel hamster was housed on the opposite side, separated by a divider consisting of two perforated metal panels spaced 1 cm apart. After 24 h of exposure, the sentinel hamsters were removed and housed individually. The WT/D614G, Alpha and Delta variants were transmitted efficiently to sentinel hamsters via the airborne route, with viral shedding first detected on 1- or 2-days post-exposure (dpe) in 100% (4/4) of the sentinel animals in each group (Fig. 1b–d). Both the donor and sentinel hamsters exhibited similar weight loss (Fig. 1e, f), reaching about 90% starting body weight on 5 dpi for donors and 5 dpe for sentinels, suggesting that both intranasal inoculation and airborne transmission caused comparable pathogenicity.

### Characterisation of airborne infectious viral emission using the Infectious Virus Transmission Tunnel

**Fig. 2:**
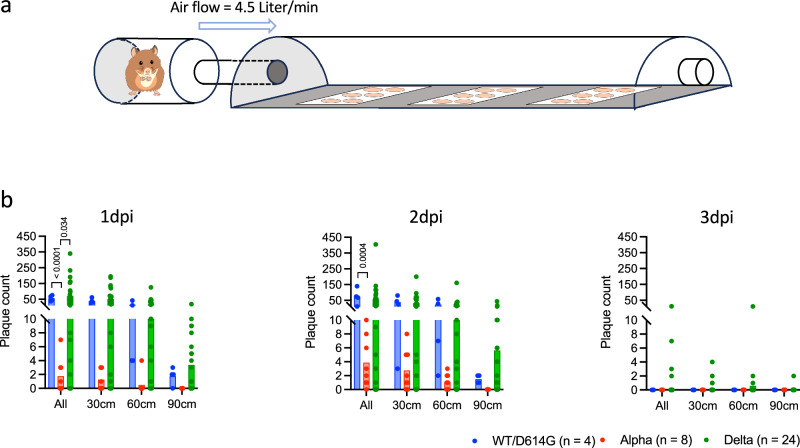
Characterisation of airborne infectious virus emission from pre-Omicron infected hamsters. **a** Schematic diagram of the Infectious Virus Transmission Tunnel (IVT). A conscious hamster was kept in the cylindrical chamber for 10 min. Air was drawn at a rate of 4.5 L/min from three ports (1.5 L/min per port) into the chamber. MDCK cell plates were placed at distances of 30 cm, 60 cm and 90 cm from the hamster. **b** Plaques collected by the IVT on 1, 2, and 3 days post-inoculation. Each dot represents one donor hamster inoculated with wildtype SARS-CoV-2 with D614G (WT/D614G, *n* = 4), Alpha (*n* = 8), or Delta (*n* = 24), while bars show the mean value.

We previously developed a unique apparatus we termed the Infectious Virus Transmission Tunnel (IVT) to quantify infectious viruses exhaled from animals infected with influenza virus and SARS-CoV-2^10,11^. Here, we used the IVT to assess the exhalation of infectious viruses from infected hamsters. The Vero E6 cells overexpressing ACE2 and TMPRSS2 were seeded on 6-well plates for viable virus detection (Fig. 2a). For WT/D614G and Alpha infected hamsters, aerosolised infectious viruses were only detected on 1 and 2 dpi, with none detected on 3 dpi, while Delta inoculated hamsters continued to exhale infectious viruses from 1 to 3 dpi (Fig. 2b). Both WT/D614G and Delta variants were detected on the third plate at 90 cm from the source, but no plaques of Alpha variant were detected at this distance. Notably, the level of infectious virus emitted from hamsters infected with Alpha variant was significantly lower than from WT/D614G (*t* test, *p* < 0.0001 on 1 dpi, *p* = 0.0004 on 2 dpi), or Delta (*p* = 0.034 on 1 dpi, *p* = 0.077 on 2 dpi) infected hamsters. However, viral RNA loads (Fig. S1a) and infectious titres (Fig. 1a) in the nasal wash samples were not significantly different among the three variants on these days. Indeed, there was little correlation between viral loads in the nose and infectious virus detected by IVT. A weak correlation was found (*R*^2^ = 0.36, *p* = 0.0020) for the Delta donors on 1 dpi, but not on 2 dpi and 3 dpi, and no correlation was found for the Alpha variant (WT/D614G was not analysed due to small sample size, *n* = 4) (Fig. S1b). Collectively, our results suggest Alpha-infected hamsters shed equivalent virus in their nose but emitted less virus into the air than Delta-infected animals, therefore, it was important to understand how the Alpha variant still transmitted efficiently via the airborne route.

### Median infective dose (ID_50_) of SARS-CoV-2 WT/D614G, Alpha and Delta to establish productive infection in the hamster model

To investigate how the Alpha variant retained efficient transmission in the hamster model despite lower emission of infectious virus, we next assessed the infectious dose required to establish productive infection for each of these SARS-CoV-2 variants. Six hamsters per group were intranasally inoculated with 100, 10, and 1 PFU of WT/D614G and Alpha variants. All hamsters became infected and shed a robust amount of infectious virus in the nasal wash samples over the following 3 days (Table 1 and Fig. S2a, b). All hamsters were euthanised on 3 dpi to examine virus titres in the lungs and bronchoalveolar lavage (BAL), and gastrointestinal tract. Infectious virus was found in the lungs and BALs of both WT/D614G and Alpha inoculated hamsters (Fig. S2c, d), with no significant difference across different inoculation doses. No infectious virus was found in intestinal samples (Fig. S2e). Maximum weight loss reached ~5% for both WT/D614G and Alpha, with no difference in clinical signs among the three dose groups (Fig. S2f, g).

**Table 1:** Titration of SARS-CoV-2 wildtype with D614G (WT/D614G), Alpha and Delta variants in the hamster model

SARS-CoV-2 variants	ID_50_	Infection rate 100 PFU	Infection rate 10 PFU	Infection rate 1 PFU	Infection rate 0.1 PFU	Infection rate 0.01 PFU
WT/D614G	<1 PFU	100% (6/6)	100% (6/6)	100% (6/6)	ND^a^	ND^a^
Alpha	0.0208 PFU	100% (6/6)	100% (6/6; 4/4)	100% (6/6, 4/4, 4/4)	75% (3/4, 2/4)	0% (0/4)
Delta	0.304 PFU	ND^a^	100% (4/4)	75% (4/4, 2/4)	25% (1/4, 0/4)	0% (0/4)

^a^*ND* not done.

To determine the smallest dose capable of causing robust infection, we next inoculated hamsters with 10, 1, and 0.1 PFU of Alpha and Delta variants, with four hamsters per group. In the 10 and 1 PFU groups, all hamsters (4/4) were infected and shed infectious virus (Table 1 and Fig. S3a, b). In the 0.1 PFU Alpha group, three hamsters (3/4) were infected, with viral shedding starting on 1 dpi, and comparable virus titres observed on day 3. In the 0.1 PFU Delta group, only one hamster (1/4) was infected, with delayed viral shedding and a half-log lower virus titre on 3 dpi. Both Alpha and Delta caused similar weight loss in the infected hamsters, reaching ~10% on 6dpi (Fig. S3c, d), regardless of inoculation dose.

To more precisely determine the ID_50_ of Alpha and Delta variants to establish productive infection, we inoculated 1, 0.1 and 0.01 PFU of each variant, in groups of 4 hamsters per dose. All hamsters (4/4) in 1 PFU Alpha groups shed virus from 1 or 3 dpi, while 50% (2/4) hamsters in 1 PFU Delta group shed virus from 2 dpi (Fig. S4a, b). In the 0.1 PFU dose group, 3 hamsters (3/4) were inoculated with Alpha shed virus from 1 dpi, and none with Delta (0/4). No hamsters in either 0.01 PFU group shed any virus. Therefore, the ID_50_ for Alpha to establish productive infection was calculated as 0.0208 PFU, which is 14.6-Fold lower than the ID_50_ of 0.304 PFU for Delta (Fig. S4e). The ratio of E gene copies to plaques was similar between Alpha (E gene copies/PFU = 7.63e3) and Delta inocula (5.71e3), eliminating that variation between different stock preparations contributed to the different ID_50_. Collectively, our data suggest that hamsters are highly susceptible to WT/D614G, Alpha, and Delta, causing productive infection in both the upper (nasal wash) and lower respiratory tracts (lung) after inoculation with very low doses. All three pre-Omicron viruses were efficiently transmitted through the air between hamsters, but interestingly Alpha variant was less efficiently emitted but retained transmissibility because it was more infectious, having a lower ID_50_.

### Duration of airborne contagiousness of SARS-CoV-2 Delta in the hamster model

**Fig. 3:**
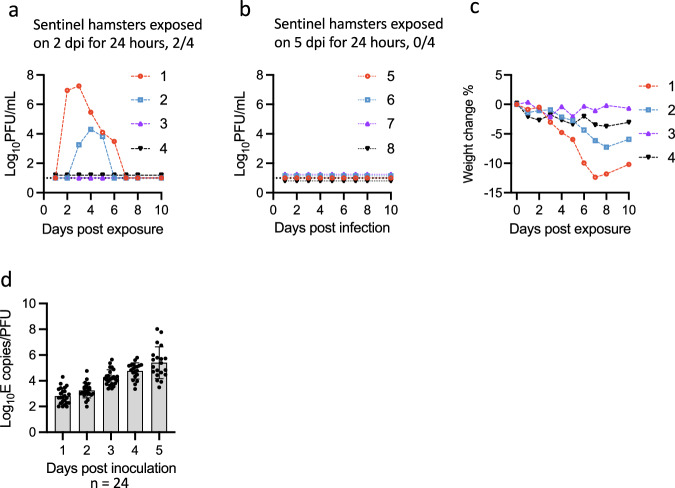
Duration of airborne contagiousness of SARS-CoV-2 Delta in the hamster model. Donor hamsters were inoculated intranasally with 100 PFU of Delta variant. During the exposure, each donor hamster was housed on one side of a custom-made ISO-cage, while a naïve sentinel hamster was housed on the opposite side, separated by a divider consisting of two perforated metal panels spaced 1 cm apart. After 24 h of exposure, the sentinel hamsters were removed and housed individually. All hamsters were sampled daily via nasal wash, and virus titres were determined by plaque assay, with a detection limit of 10 PFU/mL (dotted lines). **a**, **b** Viral shedding in nasal washes from sentinel hamsters exposed on 2 dpi (*n* = 4) (**a**) or 5 dpi (*n* = 4) (**b**). **c** Weight change in sentinel hamsters exposed on 2 dpi. Each line represents the viral shedding dynamic of an individual sentinel hamster. **d** Ratios of E gene to plaque in nasal wash samples from Delta-infected donor hamsters; mean and standard deviation are shown.

We showed above that Delta was transmitted efficiently by the airborne route following 24 h exposure of naïve sentinels at 1 dpi of donors. To investigate the duration of airborne contagiousness, sentinel hamsters were exposed to the donors via the airborne route on 2 and 5 dpi for 24 h. Two sentinel hamsters (2/4) exposed on 2 dpi were infected (Fig. 3a). The peak viral RNA titres were similar between the two infected sentinels (Fig. S5b); however, accumulated infectious virus levels were lower in one of the two positive sentinels that showed delayed viral shedding (Fig. 3a) and experienced less weight loss (Fig. 3c). In two other sentinels, viral RNA was transiently detected in nasal wash samples, but animals did not seroconvert, suggesting these animals were not productively infected (Fig. S5b). No viral RNA and infectious virus were detected in the nasal wash samples from sentinel hamsters exposed on 5 dpi (0/4) and they did not seroconvert (Figs. 3b and S5c).

The IVT was used to measure airborne viral emission on 4 and 5 dpi, but no plaques were detected (Fig. S5a), even though infectious virus could still be detected in the nasal wash samples, albeit it at several logs lower infectious titres than on day 1 and 2 (Fig. 1a). Notably, an increase in the ratio between gene copies and plaques (Fig. 3d) was observed in the nasal wash samples collected from donor hamsters as the infection progressed, suggesting the infectiousness of virus decreased, or that the later nasal washes contained significant amounts of viral RNA released from dead infected cells. Together, our results suggest that Delta-infected hamsters are most contagious early after infection, and contagiousness decreases rapidly over time. Airborne infectious viral emission, as assessed by the IVT, correlated temporally with airborne transmissibility, providing an accurate prediction for airborne transmission. In contrast, virus load in the nasal wash assessed by PCR overestimated the duration of contagiousness.

### Omicron subvariants BA.1, EG.5.1, BA.2.86 and JN.1 demonstrated varying levels of airborne transmissibility in the hamster model

**Fig. 4:**
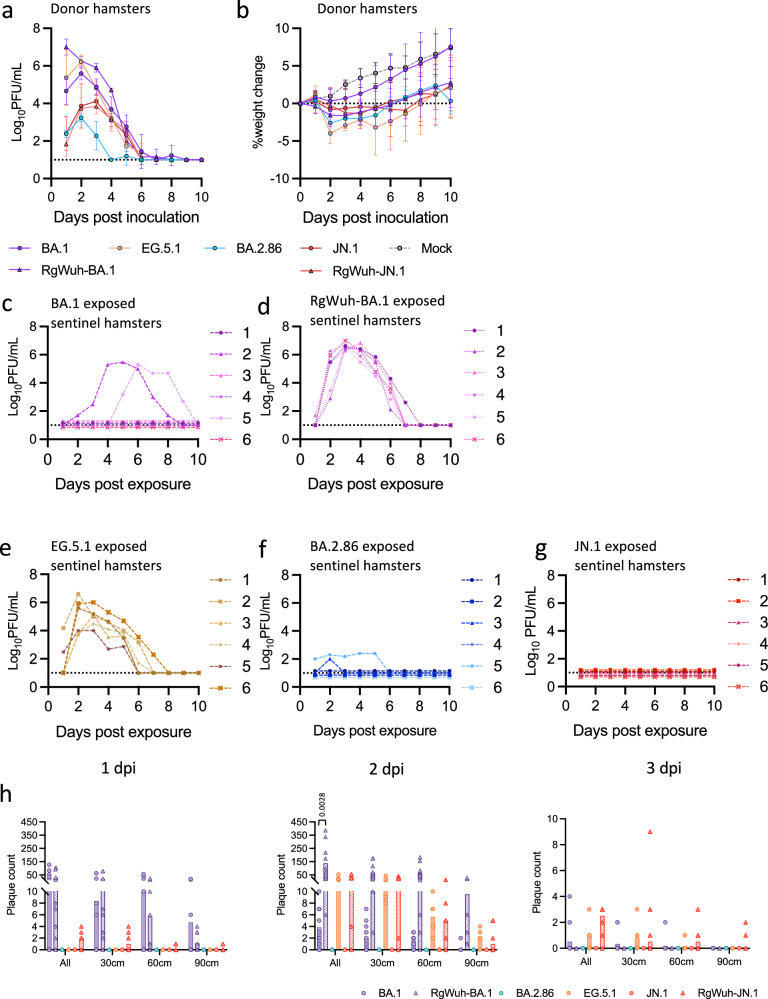
Characterisation of airborne transmissibility and infectious virus emission for omicron subvariants. **a** Viral shedding in nasal washes from donors inoculated with 100 PFU of BA.1 (*n* = 8), EG.5.1 (*n* = 6), BA.2.86 (*n* = 6), JN.1 (*n* = 6), recombinant viruses (RgWuh-BA.1 (*n* = 6) or RgWuh-JN.1 (*n* = 4), Wuhan backbone with BA.1/JN.1 Spike), or PBS (Mock infection, *n* = 4). Nasal wash samples were collected daily and viral titres were determined by plaque assay. **b** Weight change in donor hamsters. **c**-**g** Viral shedding in nasal washes from sentinel hamsters exposed to BA.1 (*n* = 6) (**c**), RgWuh-BA.1 (*n* = 6) (**d**), EG.5.1 (*n* = 6) (**e**), BA.2.86 (*n* = 6) (**f**), or JN.1 (*n* = 6) (**g**). **h** Airborne infectious virus emission from donor hamsters measured on days 1, 2 and 3 post-infection (dpi), using the IVT. Each dot represents one donor hamster, while bars show the mean value.

Previous studies have demonstrated that Omicron BA.1 (BA.1) showed reduced replication and pathogenicity in the hamster model^12^. Therefore, in our airborne transmission experiments with Omicron subvariants, sentinel hamsters were continuously exposed to donor hamsters for two weeks. Donor hamsters inoculated with 100 PFU of subvariants BA.1, EG.5.1, BA.2.86, or JN.1 showed less weight loss than seen for pre-Omicron variants and shed very different amounts of virus in their nasal wash samples. BA.1 did not cause weight loss in donor hamsters, while EG.5.1, BA.2.86, and JN.1 induced ~5% weight loss (Fig. 4b). Plaque assays showed distinct replication kinetics, EG.5.1 replicated to the highest titres, followed by BA.1, while JN.1 showed significantly lower replication, and BA.2.86 demonstrated the lowest replication with shorter virus shedding period (Fig. 4a). RT-qPCR analysis of viral genomes showed a similar pattern but with less pronounced differences in viral loads, with EG.5.1 reaching the highest titres, followed by BA.2.86, while JN.1 and BA.1 were shed at similarly low titres (Fig. S6a). Despite continuous exposure to donors with robust nasal wash titres, we found that BA.1 was still less transmissible between hamsters than pre-Omicron variants, only two of sentinel hamsters (2/6) became infected with virus shedding first detected on 2 or 5 dpe (Fig. 4c). Transient viral RNA was detected at very low levels in the nasal wash of the remaining four sentinels (Fig. S6b, c). In contrast, EG.5.1 was transmitted efficiently, 100% (6/6) sentinel hamsters were infected, with viral shedding detectable as early as 1 dpe (Figs. 4e and S6d). BA.2.86 was poorly transmitted, with only 2 out of 6 exposed sentinels becoming infected. Even then, these two animals had only low levels of infectious virus in their nasal washes (Fig. 4f and S6f, g). Neither infectious virus nor viral RNA was detected in any of the JN.1 exposed sentinel hamsters (0/6) (Fig. 4g and S6h, i). Furthermore, we also tested direct contact transmission of JN.1 between co-housed hamsters and this was also inefficient, with three of four sentinel animals becoming infected, but with delayed viral shedding from 4, 5 or 9 dpe, respectively (Fig. S7a).

To investigate whether differences in airborne transmission for this post Omicron set of variants, could at least partly be explained by differences in viral emissions into the air from donor hamsters, we measured the emitted infectious virus using the IVT (Fig. 4h). No airborne virus was detected for BA.2.86 and JN.1 variants suggesting that lack of emissions at least partly explained their poor transmission. Hamsters inoculated with BA.1 and EG5.1 emitted infectious virus, interestingly detected as early as on 1 dpi for BA.1 but not until 2 dpi for EG.5.1. A few BA.1 and EG.5.1 plaques were detectable at 3 dpi, and both variants were detected on the most distant 90 cm plate on 1 and 2 dpi respectively. Thus, it seems likely that viral emissions do not entirely explain the low transmission of BA.1.

### Recombinant viruses with SARS-CoV-2 wildtype Wuhan-hu-1 backbone and Omicron Spike protein show increased transmissibility

Two recombinant viruses with the spike gene of BA.1 or JN.1 in the genetic background of the reference strain Wuhan-Hu-1 (RgWuh-BA.1 or RgWuh-JN.1) were produced. After intranasal inoculation, RgWuh-BA.1 replicated to higher titres than full BA.1 in donor hamsters (Fig. 4a). We reasoned that the increased early viral shedding in nasal wash might translate into the virus being more readily transmitted through the airborne route. Indeed, 100% sentinels (6/6) (compared to 2/6 for wildtype BA.1, Fig. 4c, d) were infected and shed robust levels of virus from 1 or 2 dpe (Figs. 4d and S6j). As seen in RgWuh-BA.1 infected donors, there was marginal weight loss in the sentinel animals (≤ 5%) (Figs. 4b and S6k). Airborne viral emission from the RgWuh-BA.1 donors started on 1 dpi and peaked on 2 dpi, with significantly higher levels of infectious virus detected in emitted air compared to wildtype BA.1 on 2 dpi (*t*-test, *p* = 0.0028) (Fig. 4h). An increase in direct contact transmission was observed for a recombinant virus with the RgWuh-JN.1. RgWuh-JN.1 was transmitted efficiently to direct contact sentinels (100%, 4/4), with viral shedding detectable from 2 dpe (Fig. S7b). Interestingly, the level of viral shedding was not increased in the nasal wash samples from donor hamsters inoculated with RgWuh-JN.1 over that seen for wild type JN.1 (Fig. S7c), but in the IVT assay, airborne infectious viral emission was now readily detected, starting on 1 dpi and peaking on 2 dpi at titres comparable to RgWuh-BA.1 (*t*-test, *p* = 0.074) (Fig. 4h). These findings show that viral genes other than the spike contribute to the attenuated replication and transmissibility of BA.1 and JN.1 observed in the hamster model but that recombinant viruses with post-Omicron spike could be used in preclinical models to assess interventions based on spike.

## Discussion

Airborne transmission of respiratory viruses is a complex process. Understanding the characteristics of each step in the transmission process for various variants can elucidate how SARS-CoV-2 evolves to balance overcoming existing immunity while maintaining efficient airborne transmission. In this study, using the hamster model, we demonstrated that pre-Omicron variants, including the WT/D614G, Alpha, and Delta variants, are highly infectious to hamsters and transmissible via the airborne route. Although the Alpha variant exhibited reduced aerosolisation from infected donors, this was compensated for by a lower ID_50_. Thus, Alpha variant transmission was still efficient because the lower amount of infectious virus that reached the sentinel animals was more capable of initiating infection. We also showed that Delta-inoculated hamsters were most contagious early during the infection course, with airborne infectiousness decreasing rapidly, consistent with less airborne viral emission assessed by the IVT, and correlating with an increase in particle:PFU ratios in the nose of donor animals at later times post-infection. Finally, we found that the Omicron subvariants showed varying levels of airborne transmissibility between hamsters, which also largely correlated with airborne viral emissions measured by the IVT. By using recombinant viruses with Omicron spike proteins, we demonstrated that mutations outside the spike protein contribute to differences in virus aerosolisation and onward airborne transmissibility in hamsters.

While various experimental settings are employed across different laboratories to study the airborne transmission of SARS-CoV-2, pre-Omicron variants consistently show efficient airborne transmissibility, and the Alpha variant has a lower infectious dose and outcompeted the wildtype strain in the hamster model^6–10^. However, these studies did not assess infectious virus exhaled into the air. Although studies of naturally infected people reported that viral shedding in exhaled breath aerosols was significantly greater during infections with Alpha as well as Delta and Omicron than with wildtype strains, these studies measured RNA and not infectious virus^14^. We show here that viral RNA load is not the best indicator of contagiousness, and the ratio of RNA to infectious virus varies over the time course of infection. Other differences between our hamster study and human studies are anatomical differences, ACE2 receptor sequence and the complex immunity statuses of the infected and/or vaccinated humans compared to immune naïve animals.

Although hamsters have been reported to be highly susceptible to SARS-CoV-2 variants^6,8^, the ID_50_ of SARS-CoV-2 has rarely been reported. To ensure reproducible infection in hamsters, most groups have used inoculation doses exceeding 1000 PFU (or TCID_50_). Rosenke et al. reported an ID_50_ of 5 TCID_50_ for the wildtype SARS-CoV-2^15^. Here, we calculated the ID_50_ of the Alpha (0.0208 PFU) and Delta (0.304 PFU) variants, which were much lower than expected. This finding may explain why these strains of SARS-CoV-2 can be transmitted so efficiently in hamsters. Notably, the virus stocks used in this study were propagated and titrated using the Vero cells overexpressing ACE2 and TMPRSS2, which are more susceptible to SARS-CoV-2 than standard Vero cells. This methodological difference may partially explain lower ID_50_ determined in this study. In addition, to avoid the bias in virus stock preparation, we deliberately selected Alpha and Delta stocks with a similar ratio of viral RNA to infectious virus, a factor that has been neglected in previous studies. The ID_50_ for productive infection established in this study will support future SARS-CoV-2 research, particularly for studies requiring robust virus shedding. Importantly, using a lower infectious dose in animal studies (such as 100 PFU or even 10 PFU) may better mimic the dynamics of the infection process^16^, as we observed in SARS-CoV-2 human challenge studies^17,18^.

In our transmission studies, we introduced sentinel hamsters at various timepoints following infection of donor animals with the highly transmissible Delta variant. Our results demonstrated a clear temporal correlation between infectious airborne viral emissions (measured by IVT) and actual airborne transmission (assessed using sentinel animals). For Omicron BA.1, JN.1, and BA.2.86, we observed low transmission efficiency even with continuous exposure, therefore, we did not proceed to perform transmission experiments by using sentinel animals exposed at different timepoints. In contrast, EG.5.1 exhibited distinct transmission dynamics: both viral shedding and airborne emissions peaked at 2 dpi, with two sentinel infections detected as early as 1 dpi (2/6 animals), and all sentinels were infected by 2 dpi (6/6 animals) (Fig. 4e). Notably, IVT sampling detected minimal infectious virus on 3 dpi, indicating that airborne transmission occurs primarily during early infection. Despite detecting infectious virus and high viral RNA loads in nasal wash samples on 4–5 dpi, no infectious virus was aerosolized or exhaled during these later timepoints. Correspondingly, we observed no transmission events on 5 dpi. These findings align with other hamster model studies demonstrating that SARS-CoV-2 contagiousness peaks early in infection, with aerosol transmission ceasing by 3 dpi^6,7^. Our results highlight the utility of IVT for accurately determining the duration of contagiousness, as opposed to upper respiratory tract viral detection, which may overestimate transmission potential. These data provide evidence for establishing evidence-based quarantine periods for SARS-CoV-2-infected patients.

We observed an increasing ratio of viral RNA to infectious virus in nasal wash samples as infection progressed. This finding aligns with a household contact surveillance study of SARS-CoV-2-infected participants, which similarly reported a temporal increase in the RNA-to-infectious-virus ratio among infected contacts^19^. Indeed, a correlation between virus titers in the upper respiratory tract and viral emission into the air was only found on 1 dpi. As infection advances, innate and adaptive immune response products may interfere with either infectious virus particle assembly or aerosolisation^20^. Notably, these immune modulators may become incorporated into exhaled aerosol particles. When deposited in a new host’s respiratory tract, they could potentially modulate establishment of infection. Previous studies have shown that recombinant interferon sprays can prevent viral respiratory infections in human volunteers^21^. Investigation of aerosol components is needed to better understand how these innate immune factors dynamically contribute to transmission patterns.

Consistent with findings from other laboratories, we reported that Omicron BA.1 exhibits compromised replication, pathogenicity, and airborne transmission in hamsters^22,23^. We showed that EG.5.1, a derivative of the XBB Omicron sublineage, regained replication and airborne transmission ability in the hamster model. Others have also observed modest to profound increases in airborne transmissibility of EG.5.1 and its precursor XBB in the hamster model^24–26^. We reported that BA.2.86 showed reduced airborne transmission, while its derivative JN.1, with the additional L455S mutation in the spike gene, did not transmit at all between hamsters. A recent study also confirmed that JN.1 fails to transmit between hamsters via contact and airborne transmission^27^. We further characterised airborne viral emission in Omicron subvariant-inoculated donor hamsters using the IVT. Airborne transmissibility between animals correlated with airborne viral emission assessed by the IVT, again, suggesting that the measurements using the IVT can be a useful complementary tool for risk assessment of new SARS-CoV-2 variants and could also reduce animal use.

Overall, since we found that Omicron subvariants were somewhat attenuated in hamsters, inducing less weight loss and replicating to lower titres, this animal model may not be optimal going forward, so we did not pursue any more ID_50_ experiments that require large numbers of animals. Nonetheless, by combining our data on viral shedding in nasal wash samples, airborne virus emissions measured via the IVT system, and transmission experiments using sentinel hamsters, we can suggest distinct strategies are also adopted by different Omicron subvariants to maintain efficient airborne transmission, consistent with our observations in pre-Omicron variants. For example, both wildtype BA.1 and EG.5.1 replicated to high titres, and showed robust levels of airborne emission on 1 and 2 dpi, but their transmission efficiency differed significantly (BA.1, 2/6; EG.5.1, 6/6). One explanation would be that BA.1 may have a higher ID_50_ than EG.5.1. Then, comparing wildtype BA.1 and recombinant RgWuh-BA.1, we observed the recombinant virus with the Wuhan virus non-spike genes displayed increased virus shedding, and altered airborne emission patterns, suggesting that higher exposure levels of sentinel animals may have contributed to the significantly higher transmission efficiency. On the other hand, comparing wildtype JN.1 and recombinant RgWuh-JN.1, we observed similar virus shedding pattern, but dramatically increased airborne emission for RgWuh-JN.1. Although airborne transmission of this recombinant was not tested, the elevated airborne emission and efficient direct contact transmission suggest improved airborne transmission efficiency. The data from recombinant viruses indicate that non-spike genes can reshape viral shedding and airborne emission profiles. Previous studies implicate nucleocapsid protein, ORF9b, and ORF6, all of which vary between original Wuhan-like virus and the post-Omicron variants, as innate immune antagonists that suppress early immune responses^3,4^. Studies using further recombinant viruses to identify key mutations in these segments, and their effects on immune evasion and transmission in ex vivo and in vivo models, would elucidate a role for such genes.

There are limitations to this study. Firstly, unlike humans, hamsters do not exhibit respiratory symptoms such as coughing or sneezing. The IVT apparatus captured infectious viruses exhaled from infected hamsters during 10 min of normal breathing. It is worth noting that even during this short period, up to hundreds of infectious virus particles were detected. This observation aligns with our human challenge study, where we did not find a strong correlation between respiratory symptom scores and viral emission^18^. Although we cannot exclude the possibility that sneezing or coughing facilitates virus-laden particles being emitted from the respiratory tract, viruses exhaled during normal breathing are clearly sufficient for onward transmission. Moreover, transmission was most efficient at early time points when clinical signs were not yet apparent. Secondly, the transmission experiment in naive hamsters does not account for pre-existing SARS-CoV-2 immunity, which is currently a significant factor in the human population. The reduced replication capacity of JN.1 in the hamster model might therefore not fully recapitulate the growth advantage JN.1 has in some human populations with pre-existing immune pressures^28^. However, since JN.1 and other post-Omicron variants seem to also contain some genetic features that confer attenuation for replication in hamsters, further studies of growth advantages will become increasingly difficult using authentic viruses in this animal model.

In summary, this study comprehensively characteries the airborne transmission process of pre-Omicron and Omicron subvariants. We observed different strategies adapted by emerging SARS-CoV-2 variants to maintain efficient airborne transmissibility. A fine balance between airborne transmission and immune evasion is essential for the virus to continue circulating in human populations. Measurements with apparatus like the IVT and quantification of infectious doses can add valuable information for better risk assessment of transmissibility of emerging new SARS-CoV-2 variants. Finally, we showed the recombinant viruses restore infectiousness and transmissibility even of contemporary antigenic variants in the hamster model and can be useful in preclinical studies of novel vaccines or antiviral interventions that target Spike protein.

## Methods

### Biosafety and ethics statement

All work performed was approved by the local genetic manipulation (GM) safety committee of Imperial College London, St. Mary’s Campus (centre number GM77), and the Health and Safety Executive of the United Kingdom, under reference CBA1.77.20.1. Animal research was carried out under a United Kingdom Home Office License, PP0142098.

### Cells and viruses

Human embryonic kidney cells (293 T; ATCC; ATCC CRL-11268) were maintained in Dulbecco’s modified Eagle’s medium (DMEM; Gibco), 10% foetal calf serum (FCS, Gibco), non-essential amino acids (Gibco), 1x penicillin-streptomycin (Gibco). African green monkey kidney (VeroE6) cells expressing human angiotensin-converting enzyme 2 (ACE2) and transmembrane protease serine 2 precursor (TMPRSS2) (VAT cells) were produced as described previously^29^. The cells were maintained in DMEM, 10% FCS, 1 mg/mL Geneticin (Gibco), 0.2 mg/mL Hygromycin B (Invitrogen). All viral stocks used in this study were grown in the VAT cells: WT/D614G (hCoV-19/England/IC19/2020, B.1.13, EPI_ISL_475572), Alpha (hCoV-19/England/205080610/2020, B.1.1.7, EPI_ISL_723001), Delta (hCoV-19/England/SHEF-10E8F3B/2021, B.1.617.2, EPI_ISL_1731019), BA.1 (hCoV-19/England/M21021166/2022, BA.1, EPI_ISL_19725384), EG.5.1 (hCoV-19/England/GT001/2023, EPI_ISL_19725385), BA.2.86 (hCoV-19/England/4040/2023, EPI_ISL_19725386), and JN.1 (hCoV-19/England/4089/2023, EPI_ISL_19725387). Two recombinant viruses with the spike gene of BA.1 (RgWuh-BA.1) or JN.1 (RgWuh-JN.1) in the genetic backbone of the reference strain Wuhan-Hu-1 were generated using reverse genetics as previously described^30,31^.

### Plaque assays

Nasal wash samples were serially diluted in DMEM and added to the VAT cell monolayers for 1 h at 37 °C. Inoculum was then removed and cells were overlayed with DMEM containing 0.2% w/v bovine serum albumin (Gibco), 0.16% w/v NaHCO3 (Gibco), 10 mM HEPES (Invitrogen), 2 mM L-Glutamine (Gibco), P/S and 0.6% Avicel (Gibco). Plates were incubated at 37 °C, 5% CO_2_ for 3 days. The overlay was then removed, and monolayers were stained with 0.05% crystal violet solution for 1 h at room temperature. Plates were washed with tap water, then dried, and virus plaques were counted. The lower limit of detection of the assay was 10 plaque-forming units per mL (PFU/mL).

### SARS-CoV-2 gene Real-time RT-qPCR

Virus genomes were quantified by Envelop (E) gene RT-qPCR as previously described^32,33^. Viral RNA was extracted from supernatants of hamster nasal wash samples using the MagMAX Viral/Pathogen Kit on the KingFisher instrument (Thermo Fisher Scientific). Real time RT-qPCR was then performed using the AgPath RT-PCR (Life Technologies) kit on a QuantStudioTM 7 Flex Real-Time PCR System. For absolutely quantification, a standard curve was generated using dilutions viral RNA of known copy number. Gene copies per ml of original virus supernatant were then calculated using this standard curve. The lower limit of detection of the RT-qPCR was 1200 copies per mL.

### Transmission experiment by using custom-made cages

Outbred male Syrian hamsters (4–6 weeks old), weighing 80–130 g, were purchased from Janvier Labs, France. Transmission studies were performed in a containment level 3 laboratory using custom-made ISO Rat900 Individually Ventilated Cages (IVC) (Tecniplast, Italy). Hamsters were randomly assigned to be donors or sentinels. In the airborne transmission experiment, a perforated metal divider was placed in the centre of the cage, separating it into two sections and allowing airflow to be directed only from the donor section to the recipient section. Donor hamsters were intranasally inoculated with 50 μl of 100 PFU of each virus while lightly anaesthetised with isoflurane. 24 h later, one donor hamster and one naïve contact sentinel hamster were housed in each section of a cage. All animals were nasal washed by consciously instilling 400 μl of PBS into the nostrils, and the expectorate was collected into a 50 ml Falcon tube. Hamsters were observed and weighed daily. Direct contact transmission experiments were conducted as described previously^10^. Briefly, a donor hamster was inoculated with 100 PFU of the virus. One day post-inoculation, two direct contact sentinels were introduced to co-housed with the donor hamster continuously with donor hamster in an ISO-cage for two weeks.

### Measuring infectious viruses exhaled from infected hamsters by using the infectious virus transmission tunnel (IVT)

The potential for SARS-CoV-2 transmission from infected hamsters was assessed using equipment designed to detect infectious virus exhaled by the animals, as previously described^10,11^. Airflow of 4.5 L/min was introduced via a bias flow pump through three ports (1.5 L/min per port) into a 10 cm (height) × 9 cm (diameter) hamster chamber. Sentinel cell (VAT cells) culture plates were placed at three distances (30 cm, 60 cm, and 90 cm) from the infected hamster. Conscious hamsters remained in the chamber for 10 min without restriction.

### Statistics

Statistical analyses were performed using Prism (Version 10.4.0). The *t*-test or one-way ANOVA was used to compare airborne viral emission, viral shedding in nasal washes, and weight change. Linear correlation analysis was used to assess the relationship between virus titres in nasal wash samples and plaques detected by the IVT. Spearman’s correlation was used to analyse the relationship between total virus shedding (area under curve, AUC) and total weight change. Virus titres were log-transformed before the analyses. For all tests, a value of *P* < 0.05 was considered significant.

## Supplementary information

Supplementary Figures

## Data Availability

Data is provided within the manuscript or supplementary information files.
