# Supplementary material for: SARS-CoV-2 variants retain high airborne transmissibility by different strategies
Source: Npj Viruses. 2025 May 1;3:39. doi: 10.1038/s44298-025-00120-1 (PMC12045995; doi:10.1038/s44298-025-00120-1)
Supplement: Supplementary file 1 — Supplementary Figures [file 44298_2025_120_MOESM1_ESM.pdf]

Supplementary Figures

Supplementary Figure 1.

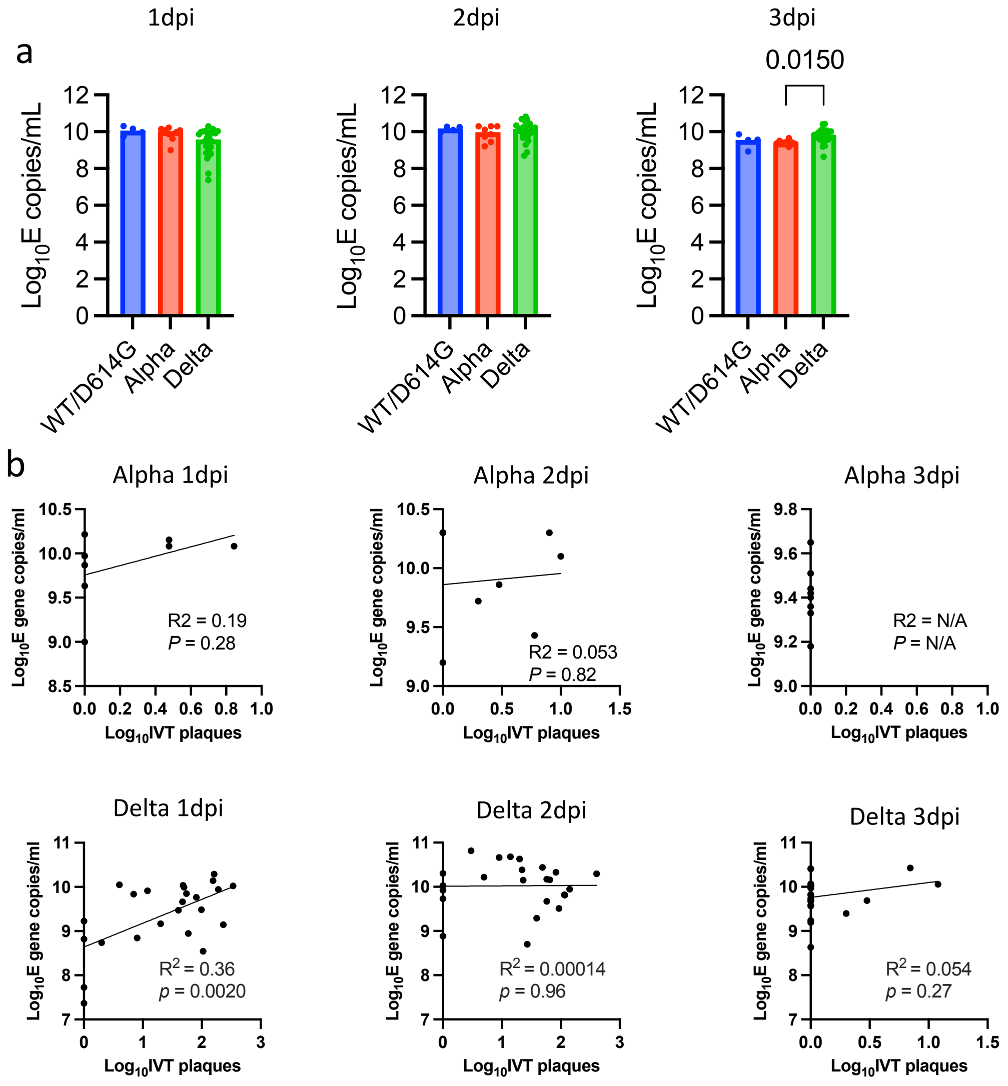

Supplementary Figure 1. Correlation of Viral Shedding in Nasal Wash Samples and Airborne Infectious Virus Emission from Pre-Omicron Variant-Infected Hamsters. Hamsters were inoculated intranasally with 100 PFU of WT/D614G, Alpha, or Delta variants. Nasal wash samples were collected daily from days 1 to 3 post-inoculation and analysed by RT-qPCR, with a detection limit of 1200 E gene copies/mL, dotted lines. The infectious virus transmission tunnel (IVT) was used to measure airborne infectious virus emission from the infected hamsters. (a) Virus titres in nasal wash samples from WT/D614G, Alpha, or Delta-infected donor hamsters. A *t*-test was used to compare virus titers between the two groups. Each dot represents one donor hamster, while bars show the mean value. (b) Correlation between viral shedding in nasal wash samples and airborne infectious virus emission from infected hamsters. R-squared and *p*-values are shown. WT/D614G was not analysed in the correlation due to a sample size of 4.

Supplementary Figure 2.

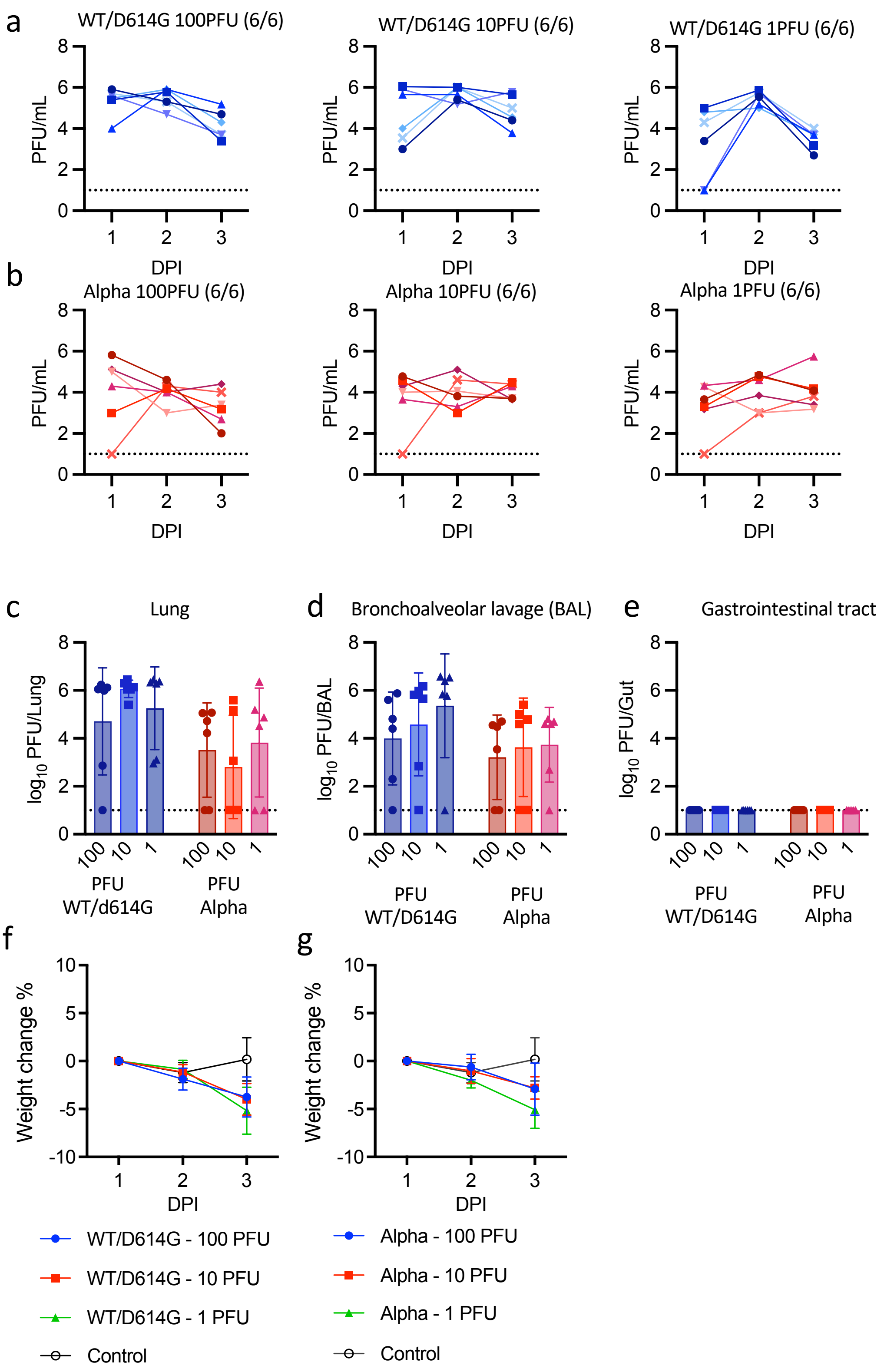

Supplementary Figure 2. Hamsters are Highly Susceptible to SARS-CoV-2 WT/D614G and Alpha Variants. Six hamsters per group were inoculated intranasally with 100, 10, or 1 PFU of WT/D614G or Alpha variant. (a, b) Nasal wash samples were

collected daily from days 1 to 3 post-inoculation and analysed by plaque assay, with a detection limit of 10 PFU/mL (dotted lines). (c-e) All hamsters were euthanised on day 3 post-inoculation to examine virus titers in the lungs (c), bronchoalveolar lavage (BAL) (d), and gastrointestinal tract (e). (f g) Weight change was recorded daily for hamsters infected with WT/D614G (f) or Alpha (g) variant.

Supplementary Figure 3.

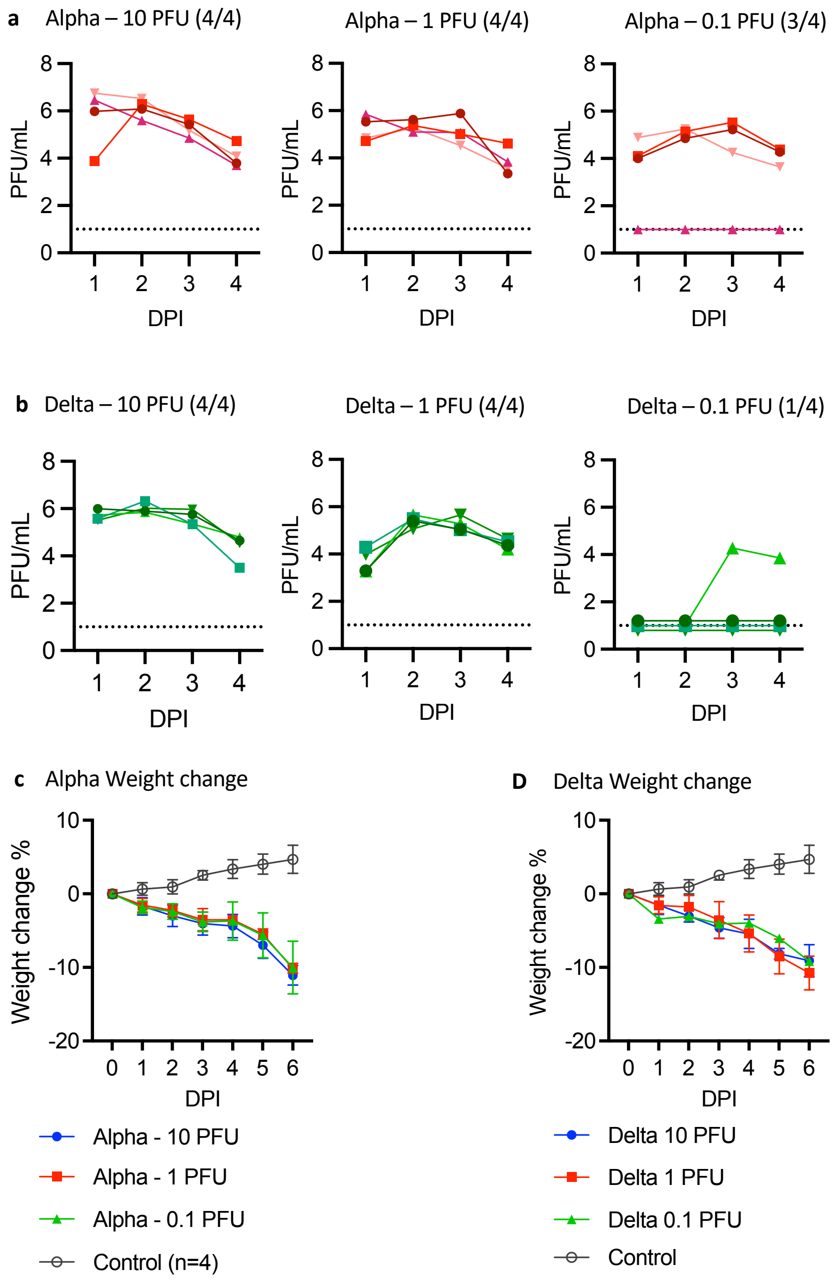

Supplementary Figure 3. Hamsters are highly susceptible to SARS-CoV-2 Alpha and Delta variants. Four hamsters per group were inoculated intranasally with 10, 1, or 0.1 PFU of Alpha or Delta variant, or with PBS (mock inoculation). (a, b) Nasal wash samples were collected daily from days 1 to 4 post-inoculation and analysed by plaque assay, with a detection limit of 10 PFU/mL (dotted lines). (c, d) Weight change was recorded daily for hamsters infected with Alpha (c or Delta (d) variant.

Supplementary Figure 4.

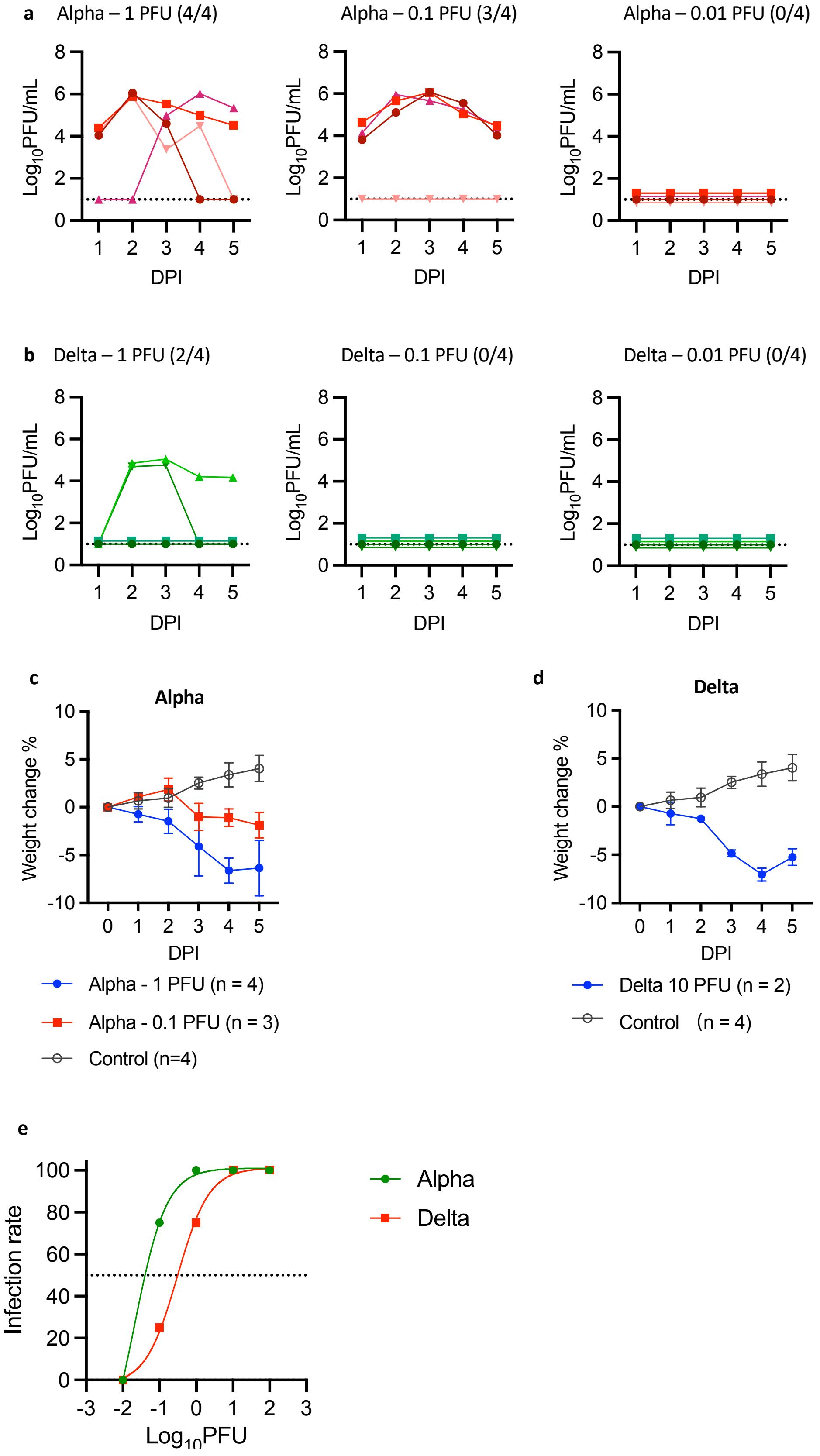

Supplementary Figure 4. Calculation of ID_{50} for SARS-CoV-2 Alpha and Delta variants in hamsters. Four hamsters per group were infected intranasally with 1, 0.1, or

0.01 PFU of Alpha or Delta variant, or with PBS (mock inoculation). (a, b) Nasal wash samples were collected daily from days 1 to 5 post-inoculation and analysed by plaque assay, with a detection limit of 10 PFU/mL (dotted lines). (c, d) Weight loss was recorded daily for hamsters infected with Alpha (c) or Delta (d) variant. (e) ID_{50} (50% infectious dose) of SARS-CoV-2 Alpha and Delta variants.

Supplementary Figure 5.

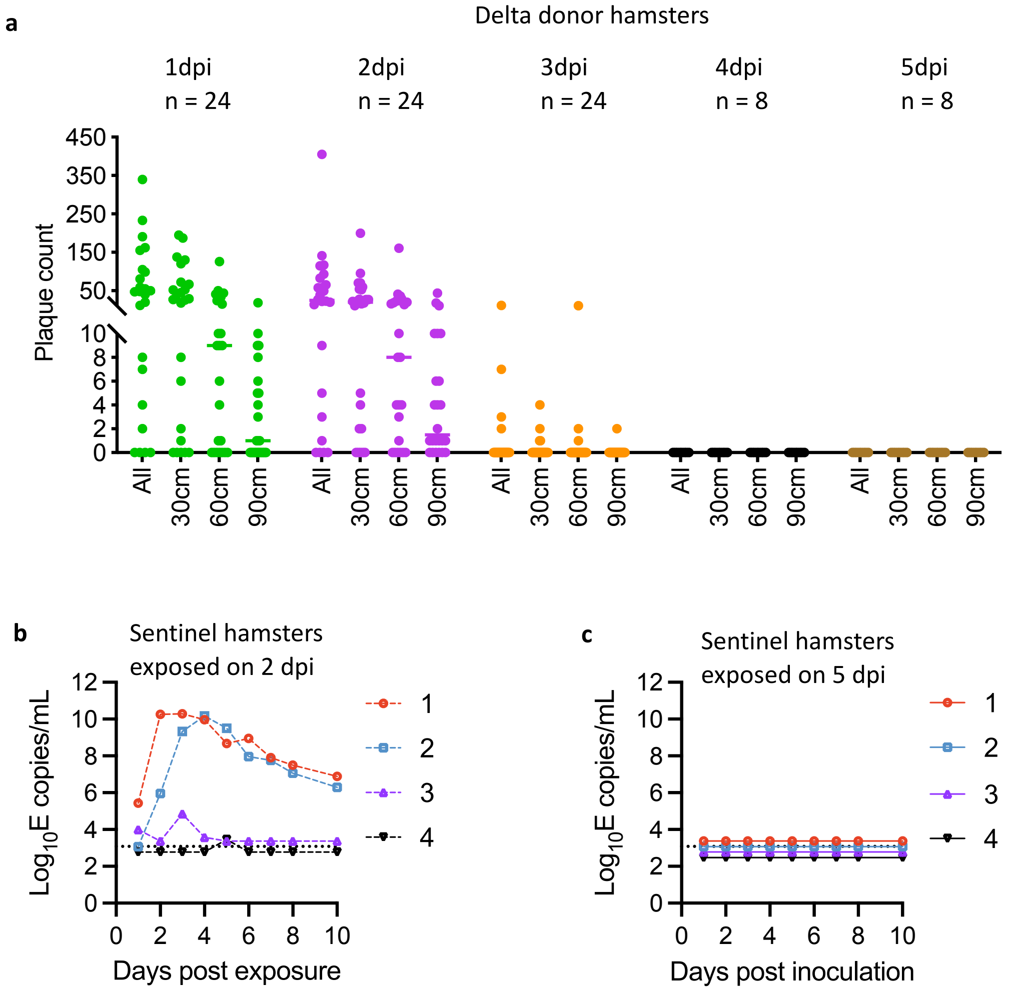

Supplementary Figure 5. Duration of airborne contagiousness of SARS-CoV-2 Delta variant in the hamster model. Hamsters were inoculated intranasally with 100 PFU of Delta variant. (a) Airborne infectious viral emission was measured daily by the Infectious Virus Transmission Tunnel from days 1 to 5 post-inoculation (dpi). (b, c) Viral shedding dynamics in nasal wash samples from sentinel hamsters exposed on 2 dpi (b) or 5 dpi (c). Nasal wash samples were quantified by RT-qPCR, with a detection limit of 1200 E copies/mL.

Supplementary Figure 6.

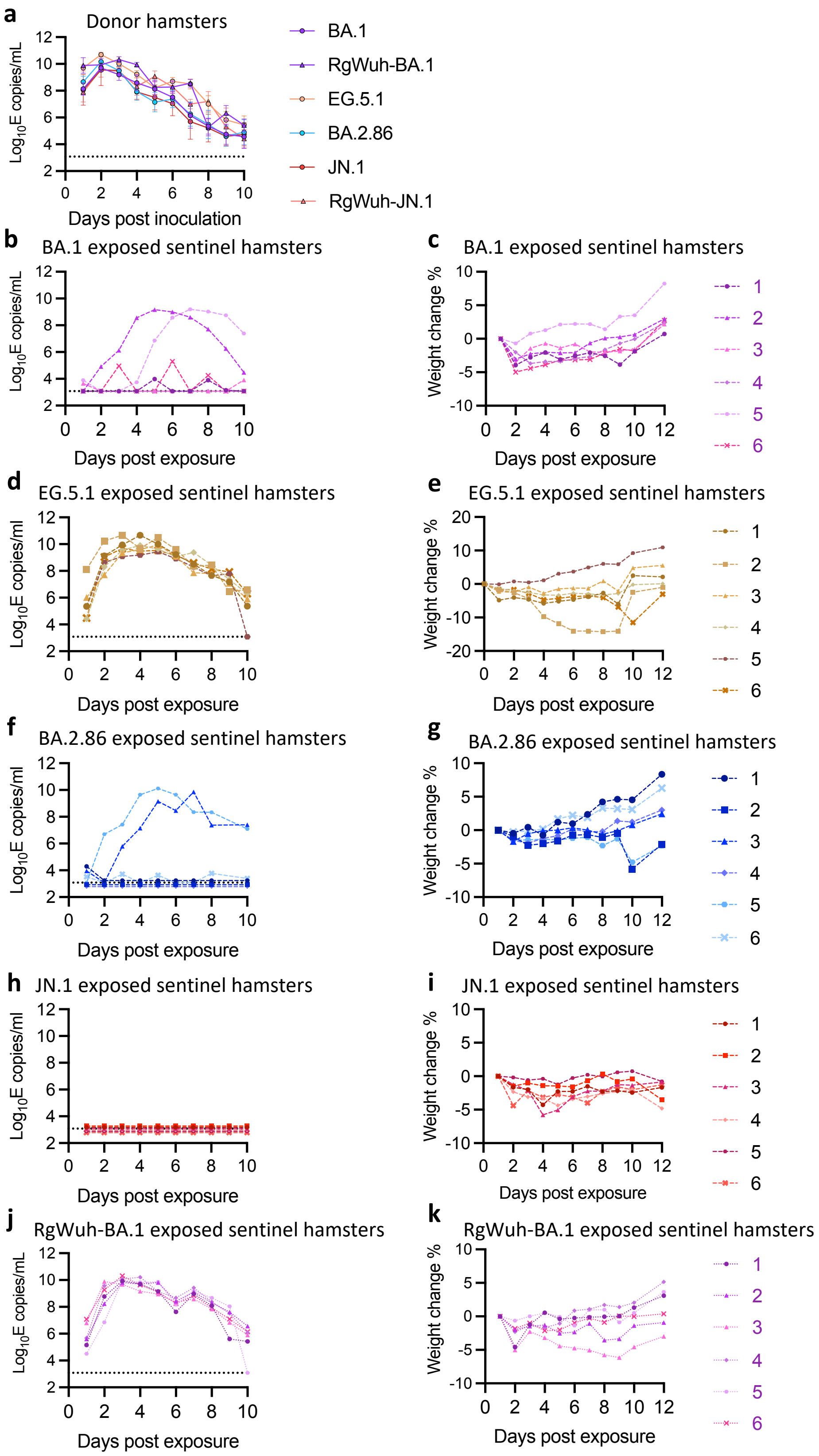

Supplementary Figure 6. Charaterisation of airborne transmission of SARS-CoV-2 Omicron subvariants in the hamster model. Hamsters were inoculated intranasally

with 100 PFU of BA.1 EG.5.1, BA.2.86, JN.1, or recombinant viruses (RgWuh-BA.1 or RgWuh-JN.1, Wuhan backbone with BA.1/JN.1 Spike). On day 1 post-inoculation (dpi), each donor hamster was housed on one side of a custom-made ISO-cage, while a naïve sentinel hamster was housed on the opposite side, separated by a divider consisting of two perforated metal panels spaced 1 cm apart. Exposure was continuous for two weeks. All hamsters were sampled daily via nasal wash and virus titres were analysed by RT-qPCR, with a detection limit of 1200 E copies/mL (dotted lines). **(a)** Viral shedding in nasal wash samples from the donor hamsters. **(b – k)** Viral shedding and weight loss in sentinel hamsters exposed to BA.1 (b, c), EG.5.1 (d, e), BA.2.86 (f, g), JN.1 (h, i) or RgWuh-BA.1 (j, k).

Supplementary Figure 7.

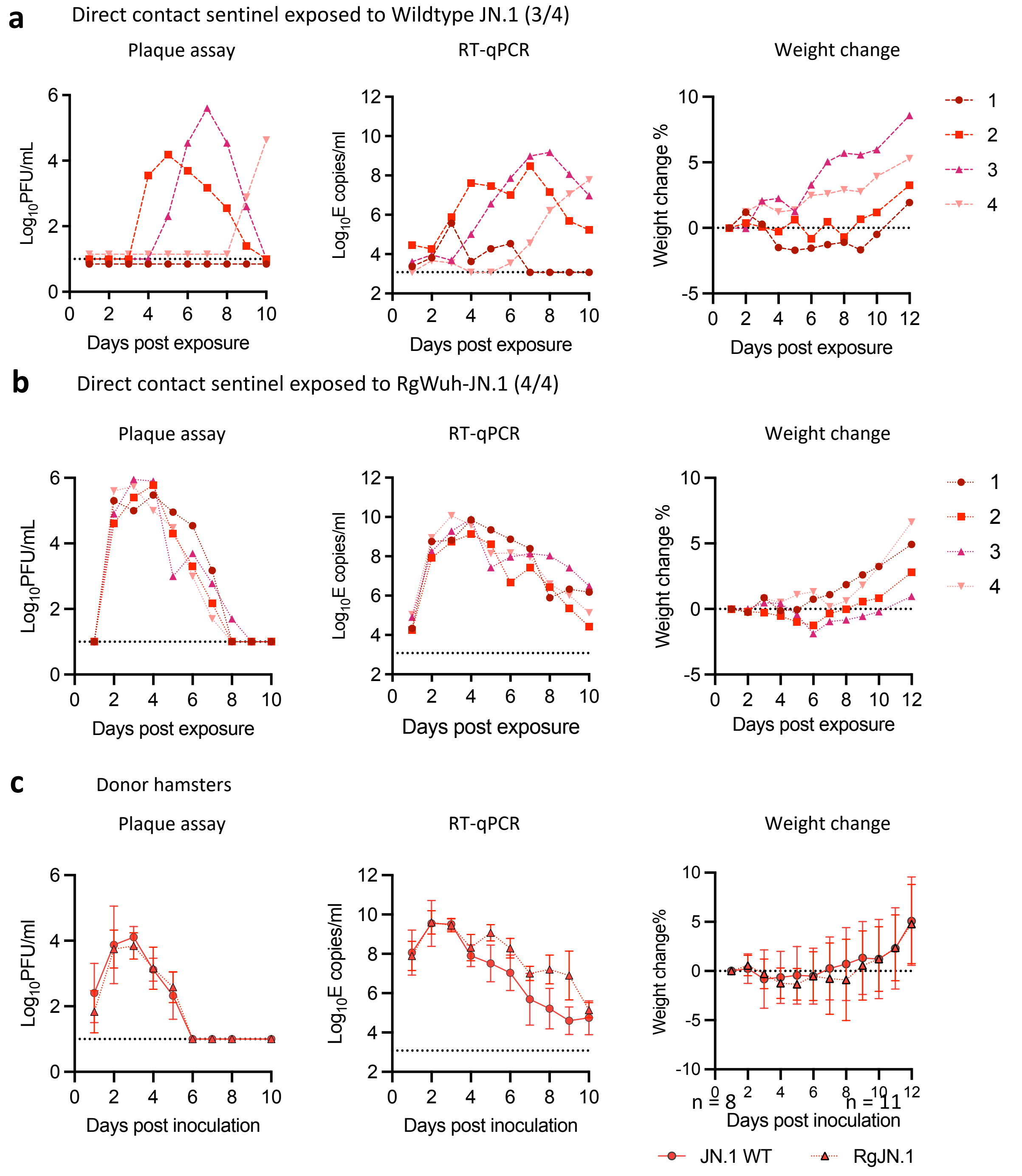

Supplementary Figure 7. Direct contact transmission of SARS-CoV-2 Omicron JN.1 and recombinant virus RgWuh-JN.1 in the hamster model. Hamsters were inoculated intranasally with 100 PFU of JN.1 or RgWuh-JN.1 variant. On day 1 post-inoculation (dpi), each donor hamster was housed with two naïve sentinel hamsters in an ISO-cage. Exposure was continuous for two weeks. All hamsters were sampled daily via nasal wash and virus titres were analysed by plaque assay (detection limit = 10 PFU/mL) and RT-qPCR (detection limit = 1200 copies/mL). **(a, b)** Viral shedding and weight changes from sentinel hamsters exposed to wildtype JN.1 (a) or recombinant virus RgWuh-JN.1

(b). (c) Comparing viral shedding and weight changes in donor hamsters inoculated with wildtype JN.1 or RgWuh-JN.1. Mean and standard deviation are shown.
